# Diffuse alveolar haemorrhage due to pheochromocytoma crisis

**DOI:** 10.1002/rcr2.722

**Published:** 2021-02-25

**Authors:** Naoki Shijubou, Toshiyuki Sumi, Koki Kamada, Yuichi Yamada, Hisashi Nakata, Hirofumi Chiba

**Affiliations:** ^1^ Department of Pulmonary Medicine Hakodate Goryoukaku Hospital Hakodate Japan; ^2^ Department of Respiratory Medicine and Allergology Sapporo Medical University School of Medicine Sapporo Japan

**Keywords:** Alveolar haemorrhage, dyspnoea, interstitial shadows, pheochromocytoma

## Abstract

Clinicians should be aware that interstitial shadows with extreme hypertension should be considered as indicators for diffuse alveolar haemorrhage due to pheochromocytoma crisis.

## Graphical abstract

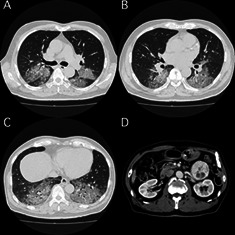

## Clinical Image

**Figure 1:**
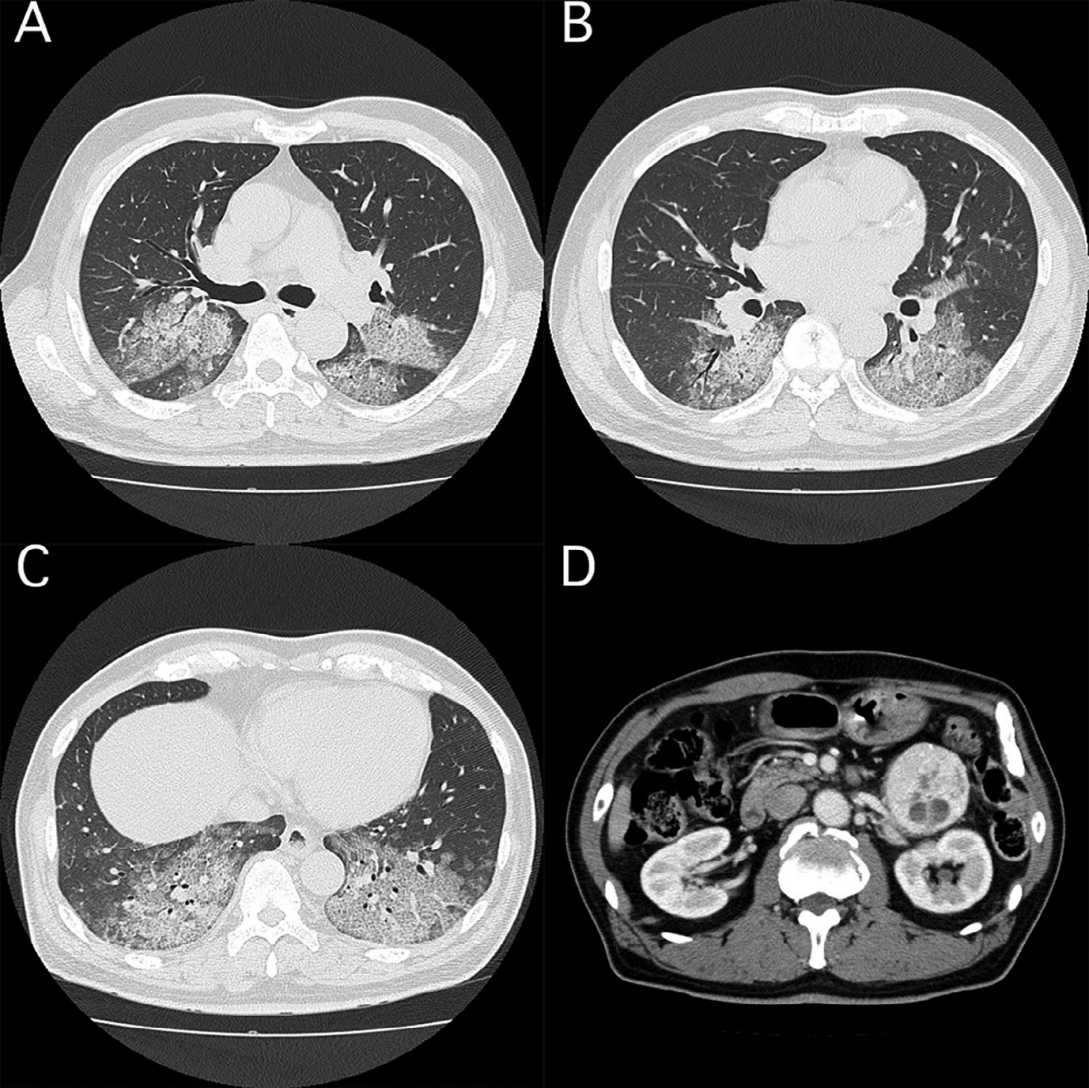
Thoracic‐abdominal computed tomography (CT) findings. CT scan showing crazy‐paving appearance (ground‐glass opacities and interlobular septal thickening) in bilateral lungs (A–C) and heterogeneous left adrenal lesion (D).

A 60‐year‐old man was referred to our hospital for dyspnoea. Thoracic‐abdominal computed tomography (CT) revealed diffuse crazy‐paving appearance and a left adrenal tumour (Fig. 1). He was put on an artificial respirator for respiratory failure. We performed bronchoalveolar lavage and obtained a gradually thickening bloody bronchoalveolar lavage fluid. He was diagnosed with diffuse alveolar haemorrhage based on the ground‐grass opacity on chest CT, and with pheochromocytoma crisis for paroxysmal hypertension and hypotension under intensive care unit management and high blood catecholamines. Imaging findings and respiratory failure both improved with anti‐hypertensive drugs and continuous haemodiafiltration for catecholamine removal. After the blood pressure settled, the left adrenal gland was excised. Pheochromocytoma crisis is a rare and life‐threatening disease and is difficult to diagnose due to a wide variety of symptoms. Autopsies of pheochromocytoma multisystem crisis with interstitial shadows, similar to this one, have been reported, where the pathology was severe pulmonary oedema and capillary microhaemorrhage [1]. Excessive secretion of catecholamine is responsible for several types of pulmonary oedema [2]; the alveolar haemorrhage in our patient may be attributed to hyper‐catecholamines due to pheochromocytoma causing capillary damage. Alveolar haemorrhage due to pheochromocytoma should be considered as a diagnosis for interstitial shadows with extreme hypertension.

### Disclosure Statement

Appropriate written informed consent was obtained for publication of this case report and accompanying images.
